# Oncological Results of Laparoscopically Assisted Radical Vaginal Hysterectomy in Early-Stage Cervical Cancer: Should We Really Abandon Minimally Invasive Surgery?

**DOI:** 10.3390/cancers13040846

**Published:** 2021-02-17

**Authors:** Aureli Torné, Jaume Pahisa, Jaume Ordi, Pere Fusté, Berta Díaz-Feijóo, Ariel Glickman, Pilar Paredes, Angels Rovirosa, Lydia Gaba, Adela Saco, Carlos Nicolau, Núria Carreras, Núria Agustí, Sergi Vidal-Sicart, Blanca Gil-Ibáñez, Marta del Pino

**Affiliations:** 1 Gynecologic Oncology Unit, Clínic Institute of Gynecology, Obstetrics, and Neonatology, Hospital Clínic Barcelona, 08036 Barcelona, Spain; atorne@clinic.cat (A.T.); jpahisa@clinic.cat (J.P.); pfuste@clinic.cat (P.F.); bdiazfe@clinic.cat (B.D.-F.); glickman@clinic.cat (A.G.); ncarreras@clinic.cat (N.C.); nagusti@clinic.cat (N.A.); 2 Institut d’Investigacions Biomèdiques August Pi i Sunyer (IDIBAPS), 08036 Barcelona, Spain; pparedes@clinic.cat (P.P.); rovirosa@clinic.cat (A.R.); lagaba@clinic.cat (L.G.); masaco@clinic.cat (A.S.); svidal@clinic.cat (S.V.-S.); 3 Surgery and Medical-Surgical Specialties Department, Faculty of Medicine, University of Barcelona, 08036 Barcelona, Spain; 4 Department of Pathology, Hospital Clínic Barcelona, 08036 Barcelona, Spain; jordi@clinic.cat; 5 Institut de Salut Global de Barcelona (ISGlobal), Faculty of Medicine, University of Barcelona, 08036 Barcelona, Spain; 6 Department of Nuclear Medicine, Hospital Clínic Barcelona, 08036 Barcelona, Spain; 7 Department of Radiation Oncology, Hospital Clínic Barcelona, 08036 Barcelona, Spain; 8 Department of Medical Oncology, Hospital Clínic Barcelona, 08036 Barcelona, Spain; 9 Radiology Department, Hospital Clínic Barcelona, 08036 Barcelona, Spain; cnicolau@clinic.cat; 10 Gynecologic Oncology and Minimally Invasive Gynecologic Surgery Unit, Department of Obstetrics and Gynecology, 12 de Octubre University Hospital, 28041 Madrid, Spain; blanca.gil@salud.madrid.org

**Keywords:** cervical cancer, laparoscopically assisted radical vaginal hysterectomy, minimally invasive surgery, laparoscopy/robotic-assisted surgery, radical hysterectomy

## Abstract

**Simple Summary:**

Some recently published studies in early-stage cervical cancer patients have shown that minimally invasive surgery (MIS), including laparoscopic and robotic approaches, might offer lower survival rates than classic open surgery. We evaluated the oncological results of a series of patients treated by laparoscopically assisted radical vaginal hysterectomy (LARVH), an infrequently used MIS technique. We included 115 patients with early-stage cervical cancer (IA1 with lymphovascular invasion, IA2, IB1, and IIA < 2 cm; International Federation of Gynecology (FIGO), 2008). The 3- and 4.5-year disease-free survival rates were 96.7% and 93.5%, respectively, and the overall survival was 97.8% and 94.8%, respectively. These survival data are comparable with those reported with the open radical hysterectomy but presented the advantages of MIS. LARVH offers excellent disease control in women with early-stage cervical cancer and can be considered as an adequate MIS alternative to open radical hysterectomy.

**Abstract:**

Background: Recent evidence indicates that some minimally invasive surgery approaches, such as laparoscopic and robotic-assisted radical hysterectomy, offer lower survival rates to patients with early-stage cervical cancer than open radical hysterectomy. We evaluated the oncological results of a different minimally invasive surgery approach, that of laparoscopically assisted radical vaginal hysterectomy (LARVH) in this setting. Methods: From January 2001 to December 2018, patients with early-stage cervical cancer were treated by LARVH. Colpotomy and initial closure of the vagina were performed following the Schauta operation, avoiding manipulation of the tumor. Laparoscopic sentinel lymph node (SLN) biopsy was performed in all cases. Women treated between 2001 and 2011 also underwent pelvic lymphadenectomy. Results: There were 115 patients included. Intraoperative complications occurred in nine patients (7.8%). After a median follow-up of 87.8 months (range 1–216), seven women (6%) presented recurrence. Four women died (mortality rate 3.4%). The 3- and 4.5-year disease-free survival rates were 96.7% and 93.5%, respectively, and the overall survival was 97.8% and 94.8%, respectively. Conclusion: LARVH offers excellent disease-free and overall survival in women with early-stage cervical cancer and can be considered as an adequate minimally invasive surgery alternative to open radical hysterectomy.

## 1. Introduction

Radical hysterectomy with pelvic lymphadenectomy is considered the standard treatment for early-stage cervical cancer (IA1 with lymphovascular invasion, IA2, IB1, and IIA < 2 cm; International Federation of Gynecology (FIGO) 2008) [1,2]. The procedure was first described using an open approach by laparotomy (Wertheim–Meigs). In the last decades, laparoscopic, and more recently, robotic-assisted laparoscopic approaches have been developed with the aim of combining similar radical excision results with the advantages of minimally invasive surgery (MIS) [3,4,5,6], which involves less blood loss, smaller scars, reduced risk of adhesions, fewer postoperative complications, and shorter hospital stay. Data from retrospective studies and several meta-analyses have indicated that recurrence and survival rates of MIS and open surgery do not differ [3,7,8,9,10,11]. Consequently, MIS approaches have progressively gained acceptance among surgeons and patients [3,4,5].

Recently, the laparoscopic approach to cervical cancer (LACC) trial, which is a well-designed, randomized, prospective clinical trial including a total of 631 patients (319 patients assigned to minimally invasive surgery arm and 312 to open surgery arm), showed that laparoscopic or robotic-assisted radical hysterectomy was associated with lower disease-free survival (DFS) and overall survival (OS) rates than open abdominal radical hysterectomy in patients with early-stage cervical cancer [12]. Subsequently, other studies and population-based surveys have confirmed the conclusions of the LACC trial, supporting the association of MIS approaches with poorer OS rates than open surgery [13,14,15].

After the publication of these studies, the guidelines developed by scientific societies have questioned the oncological safety of MIS [16]. In Europe, the European Society of Gynaecological Oncology (ESGO) has modified its former recommendations on the surgical treatment of cervical cancer, favoring open surgery as the gold standard [17], and many centers worldwide have abandoned MIS approaches, switching to the classical radical hysterectomy by laparotomy.

However, it is likely that these lower survival rates are not directly related to the MIS itself, but rather to technical procedures linked to some laparoscopic and robotic-assisted radical hysterectomy approaches, such as the use of uterine manipulators or the opening of the vagina through the abdominal cavity [12,18,19,20].

As described by Dargent [21], Coelio-Schauta or laparoscopically assisted radical vaginal hysterectomy (LARVH) combines lymphadenectomy and pelvic space creation by laparoscopy with parametrium–paracolpium resection and radical hysterectomy performed predominantly by vaginal approach, the radical vaginal hysterectomy reported by Schauta [22,23]. More recently, vaginally assisted laparoscopic radical hysterectomy (VALRH), a variation of this technique, has been described and includes transvaginal creation and closure of the vaginal cuff and laparoscopic parametrial resection [24]. The technical differences between LARVH and VALRH and other laparoscopic or robotic-assisted radical hysterectomy techniques are significant. One of the key differences is that in the LARVH and VALRH techniques colpotomy and closure of the vagina are performed at the beginning of surgery, precluding manipulation of the tumor during the procedure [25].

Nevertheless, despite these advantages [25], the implementation of LARVH has been quite limited compared with other MIS approaches, because it is considered to be a technically challenging procedure. Interestingly, the LARVH approach was not included in the LACC trial as a MIS, and therefore, was not evaluated in that trial [26].

The aim of the present report was to describe the oncological results of LARVH in a series of patients with early-stage cervical cancer.

## 2. Materials and Methods

### 2.1. Inclusion and Exclusion Criteria

From January 2001 to December 2018, all women with cervical cancer referred to the Gynecology Oncology Unit of the Hospital Clinic of Barcelona fulfilling the following criteria were included in the study: (1) histological diagnosis of cervical cancer and (2) early stage according to the International Federation of Gynecology and Obstetrics (FIGO) 2008 [27], including IA1 with lymphovascular invasion, IA2, IB1, and IIA limited to tumors <2 cm in size. The study was approved by the Institutional Review Board, and written informed consent was obtained from all subjects.

Following the protocols of our Unit [25], all women underwent LARVH and laparoscopic sentinel lymph node (SLN) biopsy. In addition, systematic pelvic lymphadenectomy was performed in all patients treated between 2001 and 2011. In 2011, SLN in early-stage cervical cancer was validated in our center. Therefore, women treated after 2011 only underwent SLN evaluation.

The exclusion criteria were as follows: (1) a medical condition precluding laparoscopy or vaginal surgery and (2) positive intraoperative SLN biopsy. Patients diagnosed with low-volume disease at final diagnosis were not excluded.

### 2.2. Laparoscopy Procedure: SLN Evaluation, Lymphadenectomy, and Radical Hysterectomy Preparation

After creating pneumoperitoneum, the laparoscope was introduced through a 12 mm umbilical port with a closed puncture technique. Four working trocars were placed: two 12 mm ports in the iliac fosses, laterally to the rectus abdominis muscle, and two 5 mm ports at the midline between the umbilicus and the left subcostal margin. Inspection of the internal genitalia, peritoneum, and the entire abdominopelvic cavity was routinely performed to exclude intraperitoneal metastases. Retroperitoneal spaces were opened and paravesical and pararectal spaces were created, delineating the uterosacral ligaments and the parametria.

Intraoperative identification of SLN was performed with preoperative lymphoscintigraphy (technetium 99 m-labeled (99 mTc) nanocolloid) and intraoperative lymphatic mapping with blue dye. Localization of the SLN was facilitated by a laparoscopic gamma probe (Navigator GPS; RMD Instruments, Watertown, MA, USA). During surgery, a lymph node was considered to be an SLN when (1) it was the first node to be visualized on lymphoscintigraphy, showed increased activity on later images, or a hot spot was seen on SPECT/CT in a different nodal area than on planar lymphoscintigraphy; (2) it was the most active node during surgery as determined by gamma tracing; or (3) it was blue. SLNs were evaluated intraoperatively by the Department of Pathology. Following clinical guidelines [28], the procedure was aborted in cases of metastatic SLN. In these cases, systematic para-aortic lymphadenectomy was performed and chemoradiation was indicated instead of radical hysterectomy.

From 2001 to 2011, the SLN procedure was being validated by our group in early-stage cervical cancer. Therefore, patients treated in this period underwent systematic bilateral pelvic lymphadenectomy after SLN dissection. Pelvic lymphadenectomy included the removal of lymph nodes of the external iliac artery, obturator fossae, and interiliac chains. From 2011 to 2018, if SLN were not found on one or both sides of the pelvis, systematic pelvic lymphadenectomy was performed uni- or bilaterally, respectively, for intraoperative evaluation.

After lymph node assessment, the uterine artery was identified and immediately divided at the origin of the hypogastric artery. The ureters were dissected distally to the ureteric tunnel, and the uterosacral ligaments were isolated. The ovaries were preserved or removed depending on the patient’s age and preferences. The round ligaments were divided, and the vesical peritoneal reflection was sectioned, separating the bladder. The trocars were left in situ to check hemostasis at the end of the procedure.

### 2.3. Vaginal Procedure: Radical Vaginal Hysterectomy

**Figure 1:**
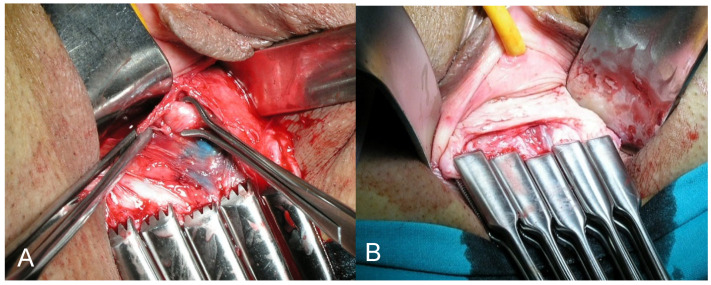
(**A**) Vaginal cuff closed with Chrobak forceps preventing tumor contact with the surgical field. (**B**) Identification of the geniculate portion of the ureter in the bladder pillar. This maneuver is facilitated by traction of the vaginal fundus using the Chrobak forceps without manipulation of the tumor.

The patient was placed in the lithotomy position. The first vaginal step was making a vaginal cuff wide enough to close it over the cervix with a disease-free margin. The vagina was incised approximately 2 to 4 cm from its fornix, depending on the tumor size and the extent of vaginal involvement (if any), closing the vaginal cuff with Chrobak forceps (Figure 1A). This procedure avoids spreading tumor cells into the abdominal cavity.

**Figure 2:**
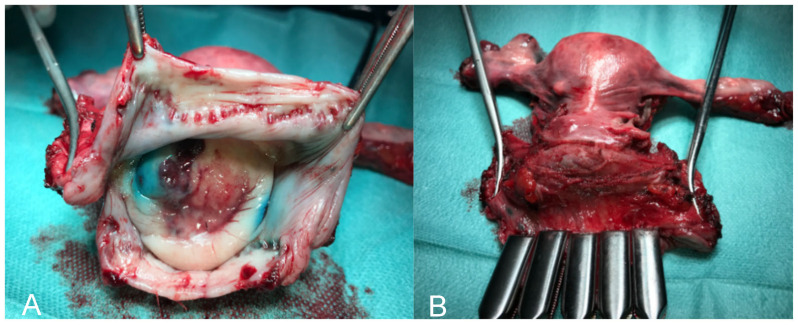
(**A**) Surgical specimen obtained at the end of surgery showing that the tumor has remained isolated throughout the intervention by closure of the vaginal cuff with the Chrobak forceps. (**B**) Image of the surgical specimen after removing the Chroback forceps. Both the cervix and tumor were undamaged and without evidence of trauma or disruption secondary to manipulation.

The bladder and rectum were dissected off the uterus at the midline, reaching the abdominal cavity. The pararectal space, previously created during laparoscopy, was entered from the vagina. The uterosacral ligaments were fully isolated and sectioned at their middle third, avoiding injury of the inferior hypogastric plexus with potential consequences to bladder, rectal, and sexual function. The paravesical space was accessed from the vagina, and the bladder pillars were isolated. The ureter was digitally identified, and the bladder pillars were divided, releasing the lowest geniculate portion of the ureter and pushing it apart (Figure 1B). The parametrium was isolated and prepared to be sectioned with the required radicality. In all cases, the surgical specimen was obtained with the Chrobak forceps closing the vagina and without any exposure of the tumor to the operative bed (Figure 2A,B). The vagina was subsequently closed with two continuous sutures. Surgical and postoperative complications, using the Clavien–Dindo classification [29], were prospectively recorded.

### 2.4. Adjuvant Treatment and Patient Follow-Up

Adjuvant postoperative treatment (radiotherapy with or without chemotherapy) was indicated following the Sedlis criteria [30], and in accordance with the National Comprehensive Cancer Network (NCCN) guidelines [16]. If indicated, a 3-dimensional image-based treatment was planned after clinical and planning target volume definition following the Radiation Therapy Oncology Group (RTOG) rules; external beam radiotherapy (EBRT) was delivered five days a week (1.8–2 Gy/d) up to a dose of 44 to 50.4 Gy [31]. Chemotherapy consisted of six weekly single doses of cisplatin (40 mg/m^2^) and was administered simultaneously with EBRT. Thereafter, brachytherapy was administered to treat the one superior third of vagina as described elsewhere.

Patients were followed every four months during the first two years, every six months up to the fifth year, and yearly thereafter. Follow-up evaluation consisted of clinical examination including Papanicolaou test and analysis of serum biomarkers (SCC, CYFRA 21.1, and CA 125) [32]. Pelvic magnetic resonance imaging was performed every six months.

### 2.5. Histological SLN Ultrastaging and Evaluation of the Lymphadenectomy Specimens

All SLNs were cut into 2 mm thick serial sections following their shortest diameter and were frozen and intraoperatively evaluated in 4 µm sections stained with hematoxylin and eosin (H&E). After intraoperative evaluation, the SLN were fixed in 10% neutral-buffered formalin and embedded in paraffin. The first 4 µm thick section was stained with H&E and examined under a light microscope. If this first section was negative, ultrastaging was performed with additional H&E and immunohistochemical sections for cytokeratin AE1/AE3 (Dako Pathology, Agilent, Santa Clara, CA, USA). Immunohistochemical studies were performed with the automated immunohistochemical system Autostainer Link 48^®^ using the EnVision system (Dako). Macrometastases were defined as metastases >2 mm in diameter, micrometastases as metastasis from >0.2 to ≤2 mm in diameter, and isolated tumor cells (ITCs) were defined as individual cells or small clusters of cells up to 0.2 mm in diameter.

Lymphadenectomy specimens were fixed in 10% neutral-buffered formalin and macroscopically dissected to isolate all the lymph nodes, which were cut into 2 mm thick sections following their largest diameter and routinely processed. Four µm thick histological sections were obtained and stained with H&E and examined under a light microscope. When present, the size of the metastasis was recorded. Isolated tumor cells and metastatic involvement below 2 mm (micrometastases) were considered as low-volume metastases.

### 2.6. Statistical Analysis

Statistical analysis was performed using IBM SPSS Statistics^®^ version 24.0. The Chi-square test or the Fisher exact test were used, as appropriate, for comparisons between categorical variables. DFS was defined as the time from the end of treatment to the diagnosis of local recurrence or metastasis. OS was defined as the time from the end of the treatment to the date of death or to the last follow-up date. Deaths by other causes not related to cervical cancer were censored at the date of death. Outcome data were evaluated by the Kaplan–Meier method, and survival curves were calculated with the log-rank test. Cox models were used to analyze the factors studied as risk factors for relapse (histology, lymph node assessment, lymphovascular space invasion, and tumor size) using the risk estimation as hazard ratio (HR) and its 95% confidence intervals (CI). A *p*-value less than 0.05 was considered statistically significant.

## 3. Results

### 3.1. General Characteristics

From January 2001 to December 2018, 115 patients with early-stage cervical cancer underwent LARVH in our center. The baseline characteristics of the patients are summarized in Table 1. The median age of the patients was 47 (range 22–76) years. One hundred eight women (93.9%) had stage IB1 disease. Sixty-six women (57.4%) were treated in the period 2001–2011 and underwent SLN biopsy followed by bilateral pelvic lymphadenectomy, and 49 (42.6%) were treated between 2011 and 2018 and underwent laparoscopic SLN biopsy as the only procedure for lymph node evaluation. No differences in terms of age, FIGO staging, tumor histology, tumor size, or lymph node status were found between women treated before and after 2011 (data not shown).

### 3.2. Surgical Results and Complications

The median operating time was 351 min (range 245–540). The median number of SLNs identified was 3.2 (range 1–8). Bilateral SLNs were identified in 75 (65.2%) patients. Ultrastaging with immunohistochemistry detected low-volume metastatic disease in SLNs in 3 cases (2.6%): one case of ITCs and two cases with micrometastases. Among the women who underwent lymphadenectomy, the median number of lymph nodes identified in the bilateral pelvic lymphadenectomy was 9.2 (range 1–30).

There was no conversion to laparotomy in any patient. Surgical complications of any grade occurred in nine women (7.8%) and seven of these women (6%) had surgical complications grade IIIb or worse according to the Clavien–Dindo classification. The median intraoperative variation in hemoglobin concentration was 2.7 g/dL (range 0.9–5.6). The intraoperative or postoperative complications and transfusion rate are shown in Table 1. Perioperative and postoperative urological complications (cystostomy, ureteral injuries, and bladder dysfunction) were the complications most frequently reported. Bladder dysfunction was more common in tumors larger than 2 cm compared with tumors of 2 cm or smaller; however, the differences were not statistically significant (13.6% [6/44] vs. 8.5% [6/71]; *p* = 0.377). The median length of the hospital stay was 5.6 days (range 2–35).

### 3.3. Follow-Up Data and Survival Outcomes

**Figure 3:**
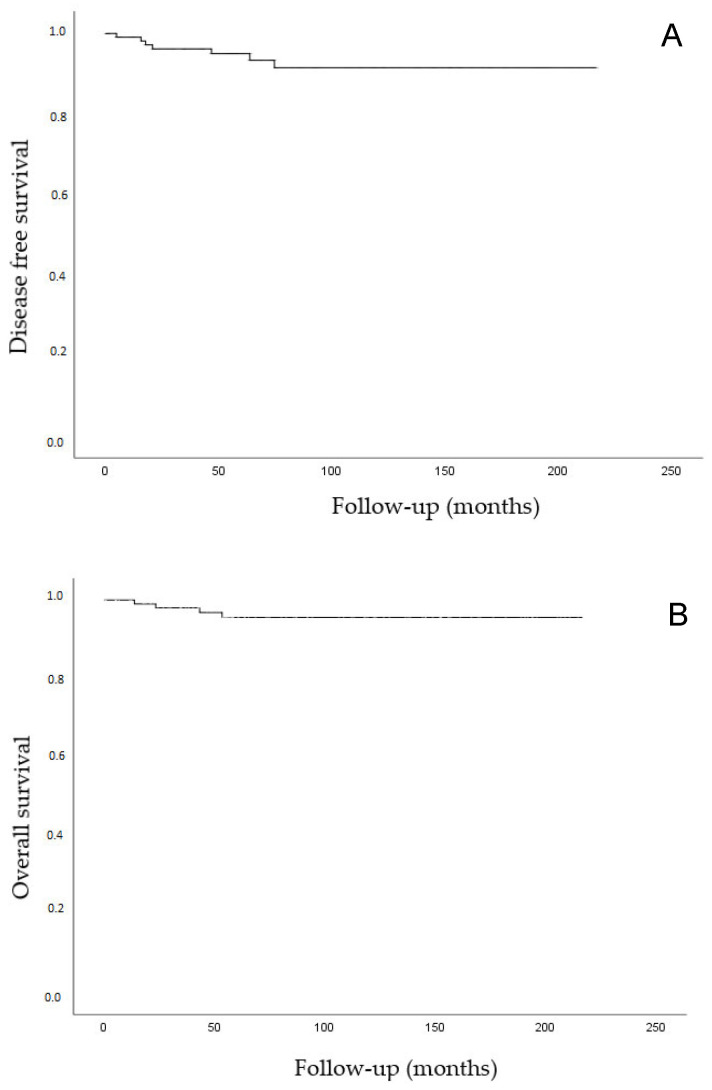
(**A**) Kaplan–Meier analysis. Disease-free survival (time from diagnosis to the first local recurrence or metastasis). (**B**) Kaplan–Meier analysis. Overall survival (time from the date of diagnosis to the date of death or to the last date of follow-up). Deaths without documented progression were censored at the date of death.

The median follow-up was 87.8 months (range 6–216 months). After surgery, 35 patients (30.4%) underwent adjuvant radiotherapy (18 EBRT and brachytherapy and 17 exclusive brachytherapy). Three patients received adjuvant chemoradiation therapy (2.6%). During the follow-up, seven patients presented tumor recurrence (recurrence rate 6.1%). Two patients (2/7, 28.6%) showed pelvic loco-regional relapse, three showed para-aortic lymph node involvement (3/7, 42.8%), and in two patients (2/7, 28.6%) distant metastases were detected (in the liver and mediastinum). No patient developed vaginal relapse. All relapses occurred within the first three years after surgery. Four patients died because of the disease (mortality rate 3.5%). The DFS rates at 3 and 4.5 years were 96.7% and 93.5%, respectively, while the OS was 97.8% and 94.8%, respectively. The DFS and OS rates are shown in Figure 3A,B.

Table 2 shows the analysis of the variables possibly associated with recurrence and mortality. None of the variables analyzed (histological type, tumor size, lymph node assessment approach, adjuvant treatment) were significantly associated with recurrence or mortality.

## 4. Discussion

In this study, women with early-stage cervical cancer treated by LARVH achieved comparable survival outcomes to those reported in the LAAC study with open radical hysterectomy [12]. Therefore, LARVH can be considered an acceptable MIS alternative to standard open surgery.

The LARVH procedure used in our study combines the laparoscopic and the vaginal approach, following the technique described by Dargent [21]. LARVH has been considered technically challenging. Indeed, radical vaginal surgery requires a longer learning curve, and consequently, more extensive training [33,34]. VALRH is a modification of the LAVRH technique introduced in some European centers to reduce perioperative and postoperative urological complications and to improve learning curves [24]. The main difficulty of LARVH approach is the exposure and visualization of critical anatomical structures and the identification of the surgical planes when the vaginal incision is performed, as some anatomic limitations such as a narrow pelvis or lack of uterine descensus may hinder the final steps of the procedure. In contrast, laparoscopic or robotic surgery closely reproduces the well-established steps of an open abdominal radical hysterectomy, and consequently, is easier to learn and perform [25]. However, in our institution, LARVH was implemented in routine practice in 1998 and the surgeons have shown that this approach is feasible, and it is possible to achieve comparable results to those of an open approach [25]. Indeed, the number of lymph nodes retrieved in this series and the surgical complications (including urinary and rectal complications and lymphedema) were comparable with the results reported in abdominal radical hysterectomy [35]. Additionally, LARVH has the advantages of other MIS: fewer scars, less risk of adhesions, reduction of postoperative complications, and shorter length of hospital stay compared with open surgery, as shown in the present series as well as in previous reports including a study published by our group [35].

Some authors have described peri- and postoperative urologic complication rates (cystostomy, ureteral injuries, and bladder dysfunction) greater than 10% [24]. Nevertheless, no significant differences were found between LARVH and abdominal radical hysterectomy in two recent meta-analyses [35,36]. Interestingly, no conversions to laparotomy and wound-related events were found in our series. While the lack of these complications might seem unusual, our results are in keeping with other reports of LARVH [35,36].

Recently, laparoscopic and robotic-assisted radical hysterectomies have been questioned as to whether they are appropriate for the treatment of early-stage cervical cancer. The results of the LACC trial showing that patients with early-stage cervical cancer treated with laparoscopic or robotic-assisted abdominal hysterectomy had a worse prognosis in terms of survival than those treated with open surgery has marked a turning point in the surgical treatment of early-stage cervical cancer [12].

Despite the controversy in relation to various aspects of the study (significant case withdrawals in both arms, gaps in histopathological data, and high rates of censored data in the LACC trial) [37] and the profound debate among professionals about a major change in their clinical practice, most cancer institutions as well as international societies have gradually encouraged abandoning MIS in favor of open surgery [16,17]. The regression of the achievements obtained by MIS would be fully justified if the surgical approach caused worse oncological outcomes.

However, it is important to remark that most of the previous studies evaluating MIS in early-stage cervical cancer were exclusively focused on laparoscopy or laparoscopic-assisted robotic radical hysterectomy [3,4,7,9,10,11,38]. These previous reports did not include other MIS approaches that combine laparoscopy and radical vaginal hysterectomy, such as LARVH, probably because these techniques are rarely performed in clinical practice due to the above-mentioned difficulties. As a result, very few centers consider radical vaginal hysterectomy as a standard approach. This results in a lack of evaluation of some MIS approaches such as LARVH, precluding the generalization of the LACC trial results to all MIS. In-depth analysis of the possible factors associated with the impaired survival outcomes is mandatory before extending these results to all MIS techniques or to other tumors as suggested by some authors [39]. Different hypotheses have been proposed to justify the worse outcomes of MIS compared to open surgery: (1) the continuous mobilization of the uterus (and the tumor) with the uterine manipulators to create the anatomical spaces required for radical hysterectomy and (2) colpotomy from the abdominal cavity with the subsequent spill of the tumor cells throughout the abdominal surgical bed [18,40]. Both factors could facilitate the lymphatic and/or hematogenous spread of tumor cells as well as their local implantation.

Remarkably, similar to the open radical hysterectomy, LARVH does not manipulate or mobilize the tumor at any time during surgery and thus, the abdomen is not exposed to tumor cells [23]. The first surgical maneuver is colpotomy and the closure of the vaginal mucosa using Chrobak forceps to isolate the tumor during all surgical procedures and prevent its exposure, fragmentation, and dissemination in the time of obtaining the surgical specimen. Another reason that has been proposed to justify the worse outcomes of the MIS is that CO_2_ gas insufflation might cause early spread of tumor cells, which in turn compromises outcomes [40,41]. However, if this were the case, the worse outcome results would be similar in other pelvic cancers, but studies comparing MIS and open surgery cystectomy or prostatectomy have not shown this impairment in survival outcomes [24,41]. According to this rationale, a recently published SUCCOR study shows that using some protective maneuvers such as avoiding uterine manipulation or carrying out the vagina’s closure over the tumors, the patients undergoing minimal invasive surgery obtained results similar to open surgery [20].

Interestingly, the survival data of the present series of patients treated by LARVH are similar to the recently reported results of the study by Köhler et al. [24], which also included patients treated by LARVH or vaginal-assisted laparoscopic radical hysterectomy, surgical approaches that also combine laparoscopy and vaginal surgery. Remarkably, the OS and DFS of both Köhler et al. and the present series are comparable with those reported with the open radical hysterectomy arm of the LACC trial which included patients with similar characteristics [12,24]. Based on these results, both LARVH and VALRH should be considered a safe oncological alternative to other MIS or open surgery. The selection of any of these surgical approaches should be performed based on the preferences and the experience in vaginal surgery of the surgical team. Table 3 shows the DFS and OS rates of the LAAC trial, the Köhler et al. study, and the present series [24].

The main strength of this study is that the surgery of all cases was performed by gynecologist oncologists with more than 20 years of experience in radical vaginal surgery. The uniformity of the procedures performed and the experience of the surgeons have probably favored the good results obtained in this study. This aspect is important since it is known that surgical results are influenced by the experience of the surgeon [42]. Moreover, all women were diagnosed, treated, and followed in the same institution, reducing possible biases due to differences in treatment or follow-up of the patients.

This study also has several limitations, one of which is its retrospective design. However, all patients were managed according to the standard protocols of our institution; thus, the treatment and follow-up of all the patients were very homogenous. Another limitation is the lack of a control group of patients treated by open surgery. However, a comparative study between LARVH and open Wertheim–Meigs carried out several years ago by our group in patients with early-stage cervical cancer showed no differences in survival outcomes [25].

## 5. Conclusions

The present study provides new data suggesting that LARVH offers high DFS and OS in women with early-stage cervical cancer. LARVH may be an alternative to the open approach in women with early-stage cervical cancer, with the advantages of minimally invasive access surgery. The advantages of LARVH related to survival prognosis, compared to other MIS (laparoscopic or robotic-assisted), should be validated in further randomized studies before definitively abandoning MIS as a treatment of early-stage cervical cancer.

## Figures and Tables

**Table 1:** Histological characteristics of the tumors and intraoperative and postoperative complications of the 115 patients included in the study. Data are shown in absolute numbers and percentages.

Feature	Number (Percentage)
FIGO classification *	
IA1 + LVSI positive	0 (0.0%)
IA2	5 (4.3%)
IB1	108 (93.9%)
IIA1	2 (1.7%)
Histological type	
Adenocarcinoma	33 (29%)
Squamous cell carcinoma	78 (68%)
Other histological types	4 (3%)
Histological grade	
Grade 1	68 (59%)
Grade 2	29 (25%)
Grade 3	18 (16%)
Lymphovascular space invasion	29 (25%)
Parametrial involvement	3 (2.6%)
Vaginal involvement	3 (2.6%)
Tumor size	
≤2 cm	71 (61.7%)
>2 cm	44 (38.3%)
Lymph node assessment	
Sentinel lymph node biopsy and bilateral pelvic lymphadenectomy	66 (57.4%)
Sentinel lymph node biopsy	49 (42.6%)
Intraoperative complications	9 (7.8%)
Incidental cystostomy	2 (1.7%)
Ureteral injury	6 (5.2%)
Bowel-rectal injury	1 (0.9%)
Conversion to laparotomy	0 (0.0%)
Transfusion requirement	9 (7.8%)
Postoperative complications	6 (5.2%)
Infection (requiring antibiotics)	5 (4.3%)
Wound infection-breakdown	0 (0%)
Venous thromboembolism	1 (0.9%)
Bladder dysfunction	12 (10.4%)

* International Federation of Gynecology and Obstetrics (FIGO) classification 2008.

**Table 2:** Analysis of the variables associated with the risk of recurrence and mortality.

Oncological Outcomes	Variables	HR	95% CI	*p*
Risk of recurrence	Histology			
	Squamous cervical cancer	1		
	Non squamous cervical cancer	0.4	0.10–2.8	0.206
	Tumor size			
	≤2 cm	1		
	>2 cm	1.2	0.6–5.93	0.824
	Lymph node assessment			
	SLN biopsy	1		
	SLN biopsy + lymphadenectomy	1.0	0.2–5.8	0.963
	Adjuvant treatment			
	No	1		
	Yes	4.2	0.8–21.5	0.080
Risk of mortality	Histology			
	Squamous cervical cancer	1		
	Nonsquamous cervical cancer	1.4	0.1–13.2	0.684
	Tumor size			
	<2 cm	1		
	>2 cm	1.5	0.2–10.9	0.668
	Lymph node assessment			
	SLN biopsy	1		
	SLN biopsy + Lymphadenectomy	0.7	0.1–7.6	0.823
	Adjuvant treatment			
	No	1		
	Yes	4.9	0.5–47.1	0.145

SLN: Sentinel lymph node.

**Table 3:** Comparison of the results of different surgical approaches in patients with early-stage cervical cancer between two previous reports including similar patients and the present study. Laparoscopic surgery/robot arm and laparotomy arm reported by the LACC trial [12], laparoscopic-assisted vaginal radical hysterectomy (LARVH)/vaginal assisted laparoscopic radical hysterectomy (VALRH) (reported by Köhler et al. [24]), and the current study data are compared. The table shows the number of patients included in each branch, the follow-up time, and the 3- and 4.5-year disease-free and overall survival.

Surgical Approach	Study	*n*	Follow-Up	Disease-Free Survival		Overall Survival	
				3 Years No. at risk (%)	4.5 Years No. at Risk (%)	3 Years No. at Risk (%)	4.5 Years No. at Risk (%)
LPS/robot arm	(LACC trial) [12]	319	30 months	87.1% 142 (45%)	86% 80 (25%)	93.8% 150 (47%)	n/a 87 (27%)
Laparotomy arm	(LACC trial) [12]	312	30 months	97.1% 134 (43%)	96.5% 90 (29%)	99% 136 (44%)	n/a 90 (29%)
LARVH/VALRH	(Köhler et al.) [24]	389	99 months	96.8% 305 (78%)	95.8% 271 (70%)	98.5% 306 (78%)	97.8% 273 (70%)
LARVH	(present study)	115	87.8 months	97.7% 86 (77%)	93.5% 65 (58%)	97.8% 88 (79%)	94.8% 66 (59%)

## Data Availability

The data presented in this study are available on request from the corresponding author. The data are not publicly available due to privacy restrictions.
